# Observations on Medical Reform

**Published:** 1806-06

**Authors:** 


### *To the Editors of the Medical and Physical Journal.*

Gentlemen,

As the spirit of reform in our profession is abroad, and legislative interference is requested, to prevent the uneducated from administering to the sick at the peril of their lives; it becomes us to be very circumspect in the choice we make of persons whose opinion is to decide on the quantum and kind of knowledge, necessary to qualify for the different departments of our profession. I see, it is proposed to invest the universities with additional privileges, to inquire into the practitioners of their districts. To this I object, at least as far as it concerns the University of Cambridge; for though their statutes contain most excellent regulations for the education of medical students, its practice is at variance with its most positive institutions. The statute* that relates to medical degrees, contains five clear and determinate regulations; and though they have lately enforced a restriction,† which it does not contain, only

---

* Medicinæ studiosus sex annos rem medicam discet ejus lectionis auditor assiduus; anatomias duas videat: bis respondeat: semel opponat antequam baccalaureus fiat.

† The Heads lately issued out the following; "Whereas doubts have arisen respecting the meaning of the statute." De Studiosis Medicinæ, "we, the under-written, are of opinion, and do determine, that the said statute is to be thus interpreted;" that is to say, "that according to the true only one of these positive regulations is adhered to; and what is worse, that it is by the management of the University and its officers, that it is rendered impossible for a student to comply with the rest. That this is not a mere assertion, I will examine them in detail.

I. " Sex annos." This period, contrary to all the analogies of language, and to the opinion of the best interpreters of our statutes, is reduced to five years.

II. Lectionis ejus, &c." These lectures are to be given ‡ four days in the week by the Reg. Professor of Medicine, under a penalty of ten shillings a day. Considering the time at which the statute was made, the greatness of the fine proves sufficiently the importance attached to the performance of the duty. Yet these lectures the Professor refuses to give, and the penalty the Vice-Chancellor neglects to inflict.

III. " Duas anatomias videat' These dissections were formerly made by the Reg. Professor, and the students in medicine defrayed the expence.‡ The subsequent institution of the Professorship of Anatomy has, with some colour of reason, being thought to relieve the Reg. Professor from this onus. But though the present Professor of Anatomy has repeatedly desired that a certificate of attendance on his lecturess should be required, as a proof of compliance with this part of the statute, the Reg. Professor has had sufficient influence to prevent it.

IV. Bis respondeat, &c." This, it is well known, is not now required, the two acts having been reduced to one by a dispensation obtained from the King.§ But this dispensation particularly insists upon the performance of the *opponency*.

V " Semel opponat." This is uniformly prevented by the Reg. Professor; and there is not a single instance of an opponency being kept, since Sir J. Pennington filled

---

true intent and meaning of the said statute. no person can be admitted as a candidate for the degree of Bachelor in Physic, who has been habitually engaged, within the period of time prescribed by the said statute, in the practice of any trade or profession whatever." This was signed by nine of the Heads. This is called an interpretation, but it is certainly a decree. The former, the Heads can make; but to create the latter, requires the consent of the Senate, which it would never have sanctioned in this case, as it is understood to be levelled at a particular person.

|| Stat. Eliz. cap. iii. p. 227.

‡ Lib. Stat. p. 377.

¶ Lit. Reg. lib. Stat. p. 309.

 the the chair. It appears then, not only that the regulations of this statute are slighted by the University and its officers, but that the very person, who is the most active in preventing its provisions from being complied with, is the very man, who so boldly resists, what he pretends to be a violation of it. For it was to gratify Sir J. Pennington, that the new regulation, which disqualifies surgeons from proceeding in medicine, was made.

I am surprised at the supineness of the College of Physicians, at this non-observance of the statutes; for I have always understood, that some agreement was made between that body and the University, that no member of any foreign University should be made a Fellow of their College, if the latter enforced the regulations of their statutes. How far the conditions are observed, the above examination will shew.

I am, &c.

*York, May* 19, 1806. PRACTICUS.

